# Metagenomic next-generation sequencing of cerebrospinal fluid: a diagnostic approach for varicella zoster virus-related encephalitis

**DOI:** 10.3389/fcimb.2024.1509630

**Published:** 2024-12-24

**Authors:** Jin Tang, Kaimeng Wang, Haoming Xu, Jingzhe Han

**Affiliations:** ^1^ Department of General Practice, The Second Affiliated Hospital of Xi ‘an Jiaotong University, Xi’an, China; ^2^ Department of Neurology, Hebei General Hospital, Shijiazhuang, China; ^3^ Department of Geriatric Respiratory, Hebei General Hospital, Shijiazhuang, China; ^4^ Department of Neurology, Harrison International Peace Hospital, Hengshui, China

**Keywords:** varicella zoster virus, encephalitis, metagenomic next-generation sequencing, cerebrospinal fluid, diagnose

## Abstract

**Purpose:**

Varicella zoster virus-related encephalitis (VZV-RE) is a rare and often misdiagnosed condition caused by an infection with the VZV. It leads to meningitis or encephalitis, with patients frequently experiencing poor prognosis. In this study, we used metagenomic next-generation sequencing (mNGS) to rapidly and accurately detect and identify the VZV pathogen directly from cerebrospinal fluid (CSF) samples, aiming to achieve a definitive diagnosis for encephalitis patients.

**Methods:**

In this retrospective study, we analyzed the clinical characteristics and laboratory evaluations of 28 patients at the Harrison International Peace Hospital in Hebei, China, between 2018 and 2024. These patients were diagnosed with neurological disorders using mNGS techniques applied to CSF.

**Results:**

In this cohort of 28 patients, 11 were females and 17 males, with a median age of 65 (IQR: 42.3-70). VZV-RE presented with a range of clinical manifestations, the most common being headaches (81.2%), fever>38°C (42.9%), and vomiting (42.9%). Less frequent symptoms include personality changes (10.7%), speech impairments (21.4%), cranial nerve involvement (21.4%), altered consciousness (17.9%) and convulsions (3.6%). Herpes zoster rash was observed in 35.7% of the cases. Neurological examination revealed nuchal rigidity in only 5 patients. CSF analysis indicated mild pressure and protein levels increase, with all patients having negative bacterial cultures. Abnormal electroencephalogram (EEG) findings were noted in 10.7% (N=3), and encephalorrhagia on Magnetic Resonance Imaging (MRI) was observed in 3.6%. VZV-RE was confirmed through mNGS analysis of CSF within three days of admission. All patients received empiric treatment with acyclovir or valacyclovir, with 21.4% receiving hormonotherapy, and 7.14% receiving immunoglobulin therapy. At the three-month follow-up, 10.7% of the patients had persistent neurologic sequelae, and the mortality rate was 3.6%.

**Conclusions:**

Performing mNGS on CSF offers a rapidly and precisely diagnostic method for identifying causative pathogens in patients with VZV central nervous system (CNS) infections, especially when traditional CNS examination results are negative. Furthermore, the cases reported highlight the positive therapeutic effect of ganciclovir in treating VZV infections.

## 1 Introduction

Varicella zoster virus (VZV), also known as human herpesvirus type 3, is a highly contagious, neurotropic, double-stranded DNA virus, with humans as its only natural host (Bhattacharya et al., 2024). Following the initial infection, which may range from asymptomatic to causing varicella or chickenpox, VZV remains dormant in the sensory ganglia of cranial and spinal nerves (Lewandowski et al., 2024). Reactivation of VZV can lead to various clinical syndromes, most commonly herpes zoster, characterized by a dermatomal vesicular rash and neuropathic pain known as shingles. Additionally, VZV reactivation can trigger neurological complications such as meningitis, encephalitis, cerebellitis, and cranial nerve palsies (e.g., Ramsay-Hunt syndrome). Moreover, it can lead to vasculopathy, myelopathy, and retinal necrosis, underscoring the virus’s potential to affect multiple nervous system regions (Kennedy, 2023). Reactivation can occur in individuals with compromised immunity, such as the elderly or immunosuppressed, potentially leading to secondary central nervous system (CNS) infection.

Encephalitis has a high incidence and mortality rate. VZV is one of the most frequent etiologic agent of encephalitis, with or without associated herpes zoster rash (Mirouse et al., 2022). However, quick and accurate pathogen identification is the key for prompt clinical management (Chen et al., 2020). Cerebrospinal fluid (CSF) viral nucleic acid detection, including Polymerase Chain Reaction (PCR) and metagenomic Next-Generation Sequencing (mNGS), is currently the primary method for the etiological diagnosis of viral meningoencephalitis (Ramachandran and Wilson, 2020). However, PCR has limitations in clinical practice, with the process of virus isolation being labor-intensive, and there is a risk of missing detections due to the specificity of primers (Han et al., 2023). Broad-spectrum and unbiased metagenomic next-generation sequencing (mNGS) is commonly used for the nucleic acid detection of neurotropic viruses (Piantadosi et al., 2021). Since its introduction in 2014, mNGS has revolutionized diagnosing CNS infections, offering key advantages such as non-specific primer use, rapid detection, and exceptionally high sensitivity, particularly identifying previously unknown or infrequently encountered pathogens. Its application in analyzing CSF has marked a transformative shift in infectious disease diagnostics. The ability of mNGS to swiftly and accurately identify pathogens has dramatically improved the accuracy and efficiency of clinical diagnostics (Chen et al., 2024).

This study presents a summary of the characteristics of VZV-RE and proposes a mNGS detection method capable of rapidly and accurately identifying pathogens directly from CSF samples. This approach has been meticulously designed and optimized to address the challenges of diagnosing meningoencephalitis of indeterminate etiology, representing a significant advancement in clinical virology.

## 2 Materials and methods

### 2.1 Study design

This retrospective investigation was conducted at Harrison International Peace Hospital in Hebei, China, from 2018 to 2024, involving a total of 28 patients. The study population included patients who tested positive for VZV in their CSF samples using mNGS. Exclusion criteria: (1) Patients with concomitant central nervous system tumors; (2) Patients with encephalitis due to multiple pathogen infections; (3) Patients with autoimmune encephalitis; (4) Patients with incomplete clinical data. Subsequent follow-up evaluations were scheduled for the enrolled participants at one and three months post-hospital discharge.

The study was approved and conducted under the supervision of the Harrison International Peace Hospital Ethics Committee, Hengshui, Hebei (approval number: 2023109).

### 2.2 Diagnostic criteria of CNS infection

Encephalitis is diagnosed in patients with an unexplained altered mental state lasting more than 24 hours, accompanied by fever over 38°C within 72 hours, new-onset seizures, focal neurological signs, CSF pleocytosis, neuroimaging changes suggesting encephalitis, and EEG abnormalities consistent with the condition (Werner et al., 2016).

### 2.3 Data collection

Collect the following data for all enrolled patients: demographic information (age, gender), past medical history, history of alcohol use and immunosuppressive factors. Record clinical manifestations, physical examination findings, time from onset to lumbar puncture, laboratory test results (including blood and cerebrospinal fluid analysis). Obtain neuroimaging via computed tomography (CT) or magnetic resonance imaging (MRI), moreover electroencephalogram (EEG) results if performed. Analyze and assess the results of metagenomic testing in cerebrospinal fluid. Additionally, track treatment regimens(empiric antibiotics, antiviral medications, corticosteroids, immunoglobulins), complications, clinical outcomes(length of hospital stay, mortality), and follow-up results (sequelae).

### 2.4 mNGS of cerebrospinal fluid

All these patients underwent mNGS concurrently during their first lumbar puncture examination. The mNGS of CSF was meticulously carried out through the following streamlined process: (1) Specimen Collection: A volume of 1-2 mL of CSF was collected from patients via lumbar puncture, aliquoted into test tubes, and immediately stored at –80°C for 30 minutes prior to being utilized for mNGS. (2) Sample Extraction and Quality Control: Genomic DNA was extracted from the CSF samples using a micro-sample genomic DNA extraction kit (DP316, TIANGEN BIOTECH, Beijing, China). The DNA was then fragmented into 200-300 base pair (bp) fragments using a DNA cutting ultrasonic disruptor (Bioruptor Pico, Diagenode, Belgium). Following the quality control assessment of fragment sizes with a 2100 Bioanalyzer, the concentration of the DNA library was determined by quantitative PCR. (3) Library Construction: DNA libraries were constructed through a series of processes including end-repair, poly(A)-tailing, adapter ligation, and PCR amplification. Roller amplification technology was employed to amplify single-stranded circular DNA by 2–3 fold, resulting in the formation of DNA nanospheres. (4) Sequencing: The DNA nanospheres were loaded onto sequencing chips and sequenced using the BGISEQ-50 sequencing platform at the Institute of Medical Laboratory, Beijing Golden Key Gene Technology Co., Ltd., Beijing, China.

After the sequencing data underwent analysis and quality control, reads characterized by low quality, low complexity, or a length of less than 35 base pairs were discarded. The resulting high-quality sequencing data were aligned against the BWA human genome database. Subsequently, the remaining gene fragment data were compared against a microbial gene database to identify potential pathogenic microorganisms, including bacteria, viruses, and fungi. The primary indicators for analysis were the type of virus detected and the number of viral copy sequences. A positive result was defined as the detection of more than one viral copy sequence in the cerebrospinal fluid. All species included in the curated pathogen reference databases were collected from books, such as the Manual of Clinical Microbiology, Diagnosis and Illustration of Clinical Microbiology, and NCBI RefSeq genome database(ftp://ftp.ncbi.nlm.nih.gov/genomes/). Strictly only one typical high-quality representative strain whose genome sequence was downloaded from the NCBI RefSeq genome database or NCBI GenBank genome database was selected for each species. Currently, our curated database contains 12895 bacterial genomes or scaffolds, 11120 whole genome sequences of viral taxa, 1582 whole genome sequences of fungal taxa, 312 whole genome sequences of parasites, 184 mycoplasma and 177 mycobacterium.

### 2.5 Statistical methods

All data analyses were performed using SPSS 27.0 (IBM, Armonk, NY, USA). Continuous data following normal distribution were expressed as means ± standard deviations (Mean ± SD), while non-normally distributed continuous data were represented as medians with interquartile ranges (IQRs). Categorical variables were shown as counts and percentages for each category.

## 3 Results

### 3.1 The characteristics of the patients

The clinical characteristics of these 28 patients (11 females and 17 males) with VZV-RE are summarized in **Table 1**. The patients had underlying conditions such as diabetes, hypertension, and coronary heart disease, totaling eight individuals, all of whom were immunocompetent. Two patients had systemic lupus erythematosus and rheumatoid arthritis, both of whom were on long-term treatment regimens involving corticosteroids and immunosuppressive drugs, who were identified as immunocompromised patients. There were 15 individuals ≥ 65 years of age with a median age of 65 (IQR: 42.3-70 years). VZV-RE presented with a range of clinical manifestations. A herpes zoster rash was observed in 35.7% (N = 10), with 3 individuals presenting with the rash prior to the onset of symptoms. The most common neurological symptom was headache (81.2%, N = 22), subsequently vomiting and fever > 38°C (42.9%, N = 12). Additionally, some less common symptoms, such as personality changes (10.7%, N=3) and convulsions (3.6%, N = 1) were also observed. For other detailed information, refer to **Table 1**.

**Table 1:** Clinical and epidemiological characteristics of patients with neurological symptoms and VZV-mNGS-positive CSF (N=28).

Characteristic	N (%)
Female sex	11 (39.1)
Age (years), median (IQR)	65 (42.3, 70)
Underlying diseases	10 (35.7)
Immunosuppressive agents	1 (3.6)
Time from neurological symptoms onset, days, means ± standard	5.1 ± 3.5
Time from onset to lumbar puncture, days, means ± standard	6.5 ± 3.8
Time from onset to medication, days, means ± standard	5.6 ± 3.6
Fever>38°C	12 (42.9)
Headache	22 (81.2)
Herpes zoster rash	10 (35.7)
Vomiting	12 (42.9)
Personality changes	3 (10.7)
Speech impairment	6 (21.4)
Disturbance of consciousness	5 (17.9)
Cranial nerve involvement	6 (21.4)
Convulsions	1 (3.6)
Meningeal irritation	5 (17.9)

IQR, interquartile range.

### 3.2 Diagnosis of VZV-RE through mNGS

We used CSF mNGS to identify the DNA sequence of VZV. The patients’ lumbar puncture pressure values, laboratory evaluation, clinical treatments and outcome are presented in **Table 2**. The median CSF pressure was 150 mmH2O (IQR: 111.3-260mmH2O), indicating a range of pressure values in the cohort. The CSF WBC count was 109.5 × 10^6^ cells/L (IQR: 31.3-315.3 cells/L). The median CSF protein level reflecting significant variability. The CSF glucose mainly within the normal range, though some values were near the lower limit. All patients exhibited negative bacterial cultures, and no Cryptococcus, Mycobacterium tuberculosis, or other specific pathogens were identified upon examination. Abnormal electroencephalogram (EEG) results were noted in 10.7% (N = 3) of the subjects, suggesting the presence of slow-wave activity or mild abnormalities. Only one patient presented with hemorrhage involving the temporal lobe and brainstem. All patients commonly received empiric treatment with acyclovir or valacyclovir. Three individuals were administered methylprednisolone, while one individual was treated with dexamethasone for anti-inflammatory and immunomodulatory effects. One of them combined received immunoglobulin therapy for 5 days. Another patient received immunoglobulin therapy alone for 3 days, due to the detection of Gamma-Aminobutyric Acid antibodies receptor(GABAR) antibodies. At the time of hospital discharge, one patient had died. The majority of the remaining patients demonstrated full recovery during the subsequent three-month follow-up. Nonetheless, two patients persisted with symptoms of headache and dizziness. These outcomes highlight the severity of the cases and the importance of vigilant treatment and follow-up.

**Table 2:** Laboratory evaluation and clinical outcome.

Laboratory features	N (%)
CSF pressure (mmH2O), median (IQR)	150 (111.3, 260)
CSF WBC (×10^6^ cells/L), median (IQR)	109.5 (31.3, 315.3)
CSF protein (0.15-0.45mg/dl), median (IQR)	0.78 (0.61, 1.431)
CSF glucose (2.5-4.5mg/dl), median (IQR), median (IQR)	3.33 (2.78, 3.99)
CSF Chloride (120-130mmol/L), means ± standard	121.15 ± 3.69
Negative bacterial culture	28 (100)
Abnormal electroencephalogram	3 (10.7)
Encephalorrhagia	1 (3.6)
Empiric treatment with acyclovir/valacyclovir	28 (100)
Hormonotherapy	6 (21.4)
Immunoglobulin	2 (7.14)
Neurologic sequelae at 3 months*	3 (10.7)
Death	1 (3.6)

IQR, Interquartile range; *Defined as non-resolution of neurologic signs and symptoms, or general deterioration; CSF, Cerebrospinal fluid; WBC, White blood cells.

mNGS identified a number of pathogens with indeterminate clinical significance. Apart from VZV, we detected Enterococcus faecalis, Citrobacter koseri, Staphylococcus epidermidis, Cutibacterium acnes, Micrococcus luteus, and Moraxella osloensis. These findings, in conjunction with the patients’ clinical presentations and laboratory tests, are considered background contaminants without pathogenic significance. Additionally, some patients had concurrent infections with Epstein-Barr virus (EBV), Human Herpesvirus 6 (HHV-6), Hepatitis B Virus(HBV), and so on. However, a review of the clinical data for these subjects indicates that these viral findings are unlikely to explain their clinical syndromes.

## 4 Discussion

Following primary infection, VZV may reactivate, causing trigeminal neuralgia and painful dermatomal rashes characteristic of Herpes Zoster, most commonly affecting the abdominal and dorsal areas (Sun et al., 2024). Encephalitis and meningitis are the most severe complications of VZV infection, typically occurring in older individuals, those with compromised immune systems, and in association with factors such as gender, concurrent malignancies, and a history of solid organ transplantation (Yuan et al., 2023). The clinical manifestations caused by VZV infection are varied, with some patients presenting atypical symptoms. In our case series, the primary symptoms included atypical presentations such as headache, fever, and vomiting, along with mental disturbances, dysarthria, and cranial nerve damage. The absence of a rash or its delayed appearance months after the onset of symptoms leads to misdiagnosis, complicating accurate diagnosis (Dulin et al., 2024). Although reactivation of VZV is more commonly reported in the elderly (Deobhakta and Gilden, 2024), in our study, one patient was a 15-year-old immunocompromised individual with systemic lupus erythematosus (SLE) undergoing steroid therapy. Additionally, five cases (17.9%) involved patients under 50-year-old, none with immunosuppressive conditions or extraordinary medical history. These patients presented with VZV meningitis without a rash, suggesting that VZV reactivation can occur in immunocompetent young individuals, potentially spreading directly to the leptomeninges and causing aseptic meningitis. This observation aligns with the findings by Lee et al (Lee et al., 2021). The only fatal case involved a 75-year-old female with a history of diabetes and coronary heart disease. Despite long-term treatment for these chronic conditions, her immune function was normal. She was admitted with fever and mental disturbances and was initially suspected of having diabetic ketoacidosis due to elevated ketone bodies. After corrective treatment, the patient remained comatose with worsening hypoxemia. Brain MRI revealed bilateral temporal lobe necrotic foci with hemorrhagic transformation. Routine CSF analysis suggested viral encephalitis and VZV-RE was confirmed through mNGS of the CSF. Despite a month of antiviral therapy in combination with corticosteroids and immunoglobulins, the patient remained unconscious and ultimately died from multiple organ dysfunction and respiratory failure. This case highlights that prognosis is closely linked to age, underlying conditions, and the presence of cerebral hemorrhagic complications. Patients with diabetes are predisposed to diabetes-related cerebrovascular issues and tend to have poorer vascular health. Moreover, VZV-RE in elderly patients is associated with an elevated risk of cerebral hemorrhage, which significantly worsens outcomes. The involvement of cortical hemorrhage in VZV-RE may indicate a poor prognosis. Early recognition and prompt antiviral therapy are crucial for improving patient outcomes. Previous studies have shown that VZV reactivation can spread axonal spread along cranial nerve ganglia, infecting arterial adventitia and leading to vasculitis. Cerebral hemorrhage contributes to a 60% mortality rate in such cases (Wu et al., 2022).

In this study, all patients underwent lumbar puncture, revealing an inflammatory response with elevated WBC count of CSF. This confirmed the diagnosis of infectious meningitis despite negative Gram stain, India ink staining, acid-fast staining as well as CSF culture results. Conventional diagnostic methods often present challenges in identifying the causative agents of encephalitis. Although PCR detection of VZV in CSF is the gold standard for the diagnosis of VZV-RE (Kriger et al., 2024). In contrast, mNGS technology allows for the direct detection of pathogenic microorganisms from brain tissue and CSF, significantly reducing diagnostic time, particularly for patients with relapsing encephalitis. Even with extensive clinical laboratory testing, approximately 60% of acute meningoencephalitis cases remain undiagnosed regarding etiology (Wang et al., 2022). Although we did not perform PCR validation, there is literature supporting that CSF VZV-specific PCR may not increase the sensitivity for diagnosing VZV CNS infections when mNGS is conducted on CSF samples. CSF mNGS is the most sensitive microbial test for diagnosing VZV central nervous system infections and can unexpectedly identify pathogens that traditional diagnostic tests fail to detect (Zhu et al., 2021). Recently, mNGS has been utilized as a powerful tool for detecting pathogens in CNS infectious, particularly in cases involving latent or chronic viral infections such as those caused by the Herpesviridae family. This technology, with its unique ability to reduce diagnostic time to within three days (Zhang et al., 2024), was applied to all patients in our study within three days of admission. This rapid turnaround time underscores the pivotal role of mNGS in identifying pathogenic CNS infections, thereby underlining the significance of the technology.

mNGS provides the ability to identify pathogens within prior knowledge of the target. Theoretically, with sufficiently long read lengths and multiple hits to the microbial genome, NGS can accurately pinpoint the causative pathogen. Our study detected many unique reads corresponding to neurotropic viral genomes in the patient’s CSF. The advancement and application of mNGS have given medical laboratory technicians an unprecedented ability to identify the pathogens responsible for encephalitis, as mNGS detects pathogens in a target-independent manner. This technology has the potential to drastically reduce the time required for diagnosis (Wang et al., 2020). Although mNGS is a high-cost test that requires specialized equipment and laboratory infrastructure, which may limit the widespread adoption of this technology. Concurrently, the costs associated with this testing are decreasing annually due to continuous technological improvements. It is anticipated that in the near future, the costs will be within a reasonable range, facilitating its broader application in clinical settings.

We acknowledge several limitations in our study. The sample size was insufficient for epidemiological analysis and group comparisons. All subjects underwent mNGS testing only, without PCR confirmation. Further research on VZV-RE is necessary, with an emphasis on expanding the sample size and thoroughly analyzing the clinical and imaging characteristics to facilitate early diagnosis and treatment and ultimately improving patient outcomes.

## 5 Conclusion

The clinical manifestations of VZV-RE are varied, and VZV reactivation causing aseptic meningitis in immunocompetent adults, with or without herpes zoster, is more common than previously recognized. Prognosis tends to be worse in elderly patients, particularly those with cerebral hemorrhage. This study emphasizes the feasibility of using mNGS on CSF as a diagnostic tool for CNS infections. In theory, unbiased NGS can identify all potential pathogens in a single test, which is highly significant for providing rapid, accurate diagnosis and facilitating targeted antimicrobial treatment.

## Data Availability

The original contributions presented in the study are publicly available. This data can be found here: https://www.ncbi.nlm.nih.gov/bioproject/1199460.
